# Bioinformatics analysis of differentially expressed genes in hepatocellular carcinoma cells exposed to Swertiamarin

**DOI:** 10.7150/jca.33666

**Published:** 2019-10-21

**Authors:** Haoran Tang, Yang Ke, Zongfang Ren, Xuefen Lei, Shufeng Xiao, Tianhao Bao, Zhitian Shi, Renchao Zou, Tiangen Wu, Jian Zhou, Chang-An Geng, Lin Wang, Jijun Chen

**Affiliations:** 1 Department of Gastroenterological Surgery, the Second Affiliated Hospital of Kunming Medical University, Kunming, China.; 2 Department of Hepatobiliary Surgery, the Second Affiliated Hospital of Kunming Medical University, Kunming, China.; 3 Department of Critical Care Medicine, the Second Affiliated Hospital of Kunming Medical University, Kunming, China.; 4 Department of Oncology, the Second Affiliated Hospital of Kunming Medical University, Kunming, China.; 5 Mental Health Center, Kunming Medical University, Kunming, China.; 6 State Key Laboratory of Phytochemistry and Plant Resources in West China, Kunming Institute of Botany, Chinese Academy of Sciences, Kunming 650201, PR China.

**Keywords:** swertiamarin, microarray, HepG_2_ cells, hepatocellular cancer, Jun, Stat3

## Abstract

**Aim**: To explore gene expression profiling in hepatocellular carcinoma (HCC) cells exposed to swertiamarin.

**Methods**: Cell viability, apoptosis and invasion were examined in HepG2 cells after swertiamarin treatment. Tumor growth of SK-Hep-1 cells xenografted in nude mice was monitored after swertiamarin treatment. Total RNA was isolated from HepG2 cells treated with swertiamarin for microarray analysis. The data of microarray were analyzed by bioinformatics.

**Results**: Swertiamarin treatment decreased the viability and invasion while increased the apoptosis of HepG2 cells, and significantly inhibited the growth of SK-Hep-1 cells xenografted in nude mice. Pathway and biological process analysis of differentially expressed genes (DEGs) in swertiamarin treated HepG2 cells showed that PI3k-Akt was the most significant regulated pathway. 47 targets of swertiamarin were predicted by CGBVS while 21 targets were predicted by 3NN. Notably, 8 targets were predicted as the targets of swertiamarin by both programs, including two prominent targets JUN and STAT3. A large range of DEGs induced by swertiamarin could be regulated by JUN and STAT3.

**Conclusion**: Swertiamarin treatment led to significant changes in the expression of a variety of genes that modulate cell survival, cell cycle progression, apoptosis, and invasion. Moreover, most of these genes can be clustered into pathway networks such as PI3K, JUN, STAT3, which are predicted targets of swertiamarin. Further confirmation of these targets will reveal the anti-tumor mechanisms of swertiamarin and facilitate the development of swertiamarin as a novel agent for cancer prevention and treatment.

## Introduction

Hepatocellular carcinoma (HCC) is a common cancer in China and becomes the fourth leading cause of cancer related deaths worldwide 1. Despite recent developments in surgery, radiotherapy, and chemotherapy, current therapeutic approaches for HCC are still not effective. Recently, great efforts have been taken to investigate herbs with potential anti-cancer efficacy 2-4. Nevertheless, herb extracts are usually crude mixture of many compounds, and the complexity of herbs hinders the development of anti-cancer drugs. Therefore, it is important to isolate, identify and elucidate the mechanism of action of single compound from herbs as lead candidate for cancer drug discovery.

Swertiamarin (STM) is a main bioactive component in Swertia mussotii Franch (Gentianaceae), a commonly used Tibetan medicine, and exhibits broad bioactivities including hepatoprotective effects 5-7. In rat model of liver injury induced by d-Galactosamine, oral administration of STM caused significant antioxidant and hepatoprotective effects 5. Total iridoids and xanthones (TIXS) were extracted from Swertia mussotii Franch, and STM was identified as the main component of TIXS. In mouse model of alpha-naphthylisot hiocyanate induced liver damage, oral administration of TIXS significantly reduced levels of alanine aminotransferase, aspartate aminotransferase and the total and direct bilirubins 6. Furthermore, in rat model of liver injury induced by bile duct ligation, treatment with STM led to significantly decreased serum levels of serum alanine aminotransferase and aspartate aminotransferase, significantly improved liver histology, and significantly reduced inflammation and cholestasis 7. However, the efficacy of STM on HCC treatment has not been investigated.

The evaluation of anti-HCC efficacy of STM will greatly help develop novel therapeutics for HCC. To better understand the mechanism of action of STM in HCC therapy, in this study we employed microarray to screen differentially expressed genes in HepG2 hepatocellular carcinoma cells treated by STM.

## Materials and Methods

### Cell culture

HepG_2_ and SK-Hep-1 cells were cultured in Dulbecco's modified Eagle's Medium (DMEM) supplemented with 10% fetal bovine serum and 1% penicillin and streptomycin at 37℃ in a humid incubator with 5% CO_2_. STM (>95% purity) was purchased from Sigma Aldrich (St. Louis, MO, USA) and dissolved in dimethylsulfoxide (DMSO) to make stock solution at 60 mg/ml.

### Nude mouse model

Male BALB/c-nu nude mice (weight 18-22 g) were purchased from Animal Center of Kunming Medical University (No. SCXK-2015-0002) and animal experiments were approved by Animal Care and Use Committee of Kunming Medical University. Each mouse received subcutaneous injection of 3×10^6^ SK-Hep-1 cells, and when the tumor grew the mice were randomly divided into 2 groups (n=6): control group received intratumoral injection of PBS; STM group received intratumoral injection of 5 μg STM. Tumor volume was measured every week for 8 weeks, and then the mice were sacrificed and xenograft tumors were dissected to measure the weight.

### Cell viability assay

HepG_2_ cells were seeded into 96-well plates at a density of 5,000 cells/well. After overnight incubation in a humid chamber at 37˚C, the cells were treated with DMSO or different dose of STM. Viable cells were evaluated using CCK-8 Assay kit (Dojindo, Japan) according to the manufacturer's instructions. CCK-8 solution was added to the cells in 96-well plates and the plates were incubated at 37˚C for 4 h, and then 150 μL DMSO was added to each well and the plates were incubated at room temperature for 10 min. The optical density of each well was read at 450 nm using a microplate reader (Bio-Rad, Hercules, CA, USA).

### TUNEL assay

HepG_2_ cells were seeded onto coverslips in 6-well plates. After overnight incubation in a humid chamber at 37˚C, the cells were treated with DMSO or STM. The cells were fixed in 4% paraformaldehyde for 15 min, and then apoptotic cells were stained using TUNEL kit (Roche Diagnostic, Indianapolis, IN, USA) following the manufacturer's instructions. Apoptotic cells showed brown nuclear staining.

### In vitro cell invasion assay

Transwell chamber (Costar, Cambridge, MA, USA) was used to evaluate cell invasion. The upper chambers were precoated with Matrigel overnight at 4ºC. HepG_2_ cells were treated with DMSO or STM, and 100 uL of the cell suspensions were seeded in the upper chambers, while the lower chambers were filled with complete medium supplemented with 10% BSA. After 24 h incubation, the cells on the upper surface of the filters were wiped by cotton swabs and the cells on the underside of the filters were fixed, stained with crystal violet and observed under a microscope.

### Microarray

After HepG2 cells were treated with DMSO or STM, total RNA from each sample was isolated using Trizol (Invitrogen) and purified using mirVana miRNA Isolation Kit (Ambion, Austin, TX, USA) following the manufacturer's instructions. RNA integrity was determined by denatured agarose gel electrophoresis. DNA microarray was performed according to standard protocol.

CapitalBio cRNA Amplification and Labeling Kit (CapitalBio) was used to produce fluorescent dye labeled cDNA. The labeled cDNAs were then hybridized to Agilent human mRNA Array which was designed with eight identical arrays per slide (8 x 60K format). After hybridization at 42°C overnight, the arrays were washed with 2X SSC and 0.2% SDS at 42°C for 5 min, followed by washing with 0.2X SSC and 0.2% SDS at room temperature for 5 min. The array data were analyzed for data summarization, normalization and quality control by using the GeneSpring software V13 (Agilent).

### Statistical analysis

The data were presented as mean ± standard deviation (SD). Data from two samples were compared using t test and data from multiple samples were compared using single factor analysis of variance. P<0.05 was considered statistically significant.

## Results

### STM inhibits HCC

**Figure 1:**
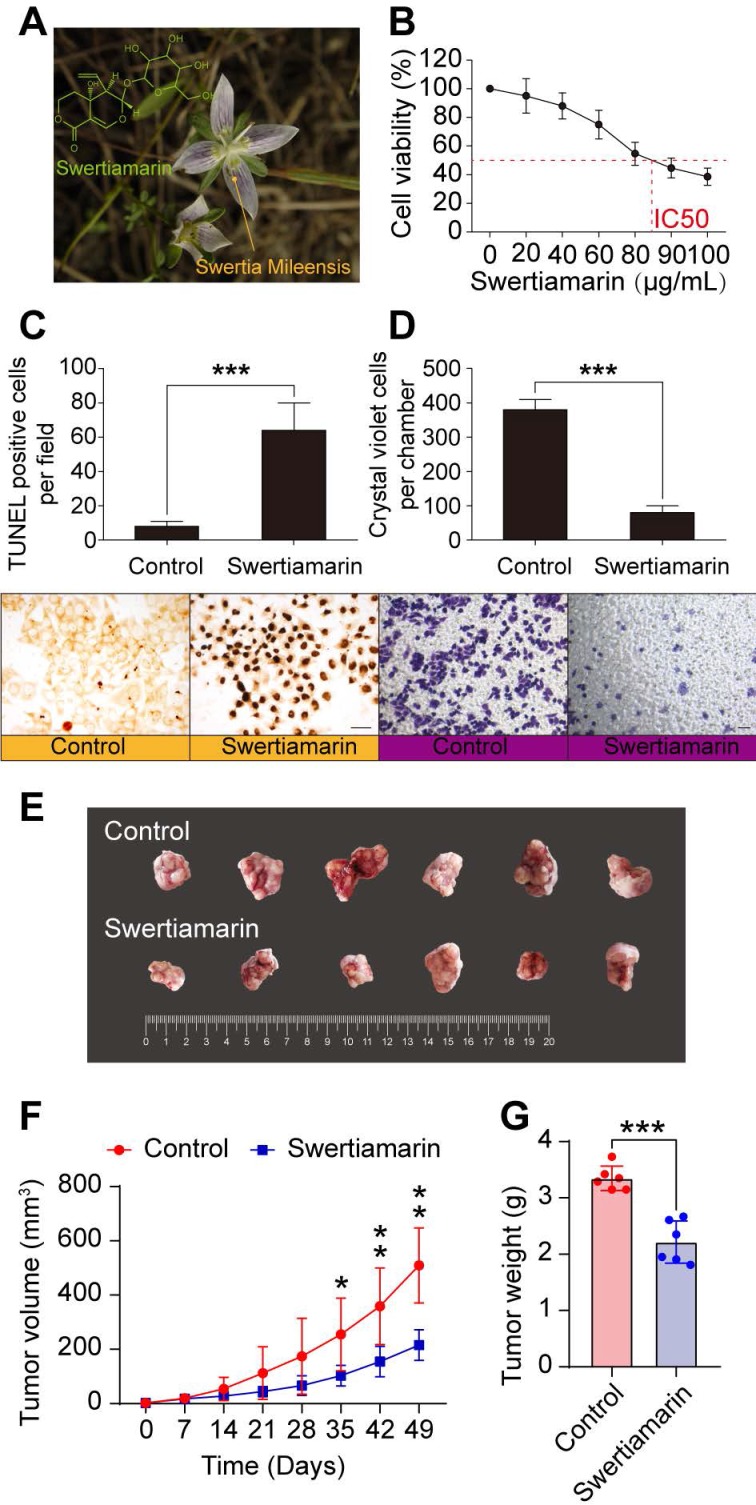
** STM inhibited HCC.** A. Chemical structure and the origin of STM. B. STM decreased the viability of HepG2 cells in a dose-dependent manner. C. STM increased the apoptosis of HepG2 cells. D. STM decreased the invasion of HepG2 cells. The data were presented as mean ± SD (n=3). *** P<0.001. Scale bar: 20 μm. E. Tumor tissues dissected from nude mice which received subcutaneous injection of SK-Hep-1 cells. F. Tumor growth curves of each group of nude mice which received subcutaneous injection of SK-Hep-1 cells. G. The weight of tumor tissues dissected from nude mice nude mice which received subcutaneous injection of SK-Hep-1 cells. The data were presented as mean ± SD (n=6). *P<0.05, **P<0.01, ***P<0.001.To select the differentially expressed genes, we used threshold values of ≥2 and ≤-2-fold change and Benjamini-Hochberg corrected p value of 0.05. The data were Log2 transformed and median centered by genes using the Adjust Data function of CLUSTER 3.0 software. Finally, tree visualization was performed using Java Treeview (Stanford University School of Medicine, Stanford, CA, USA).

The origin and the structure of STM were shown in Fig. 1A. STM inhibited the viability of HepG2 cells in a dose dependent manner (Fig. 1B). Moreover, STM significantly increased the apoptosis of HepG2 cells compared to vehicle treated cells (Fig. 1C). Furthermore, STM significantly inhibited the invasion of HepG2 cells compared to vehicle treated cells (Fig. 1D). To further confirm that STM inhibits HCC, we chose poorly differentiated and more malignant HCC cell line SK-Hep-1 to establish in vivo HCC model. We found that STM significantly inhibited the growth of SK-Hep-1 derived tumor in nude mice (Fig. 1E, F, G). Collectively, these data demonstrate that STM inhibits HCC.

### Microarray analysis of differential genes in HepG2 cells exposed to STM

**Figure 2:**
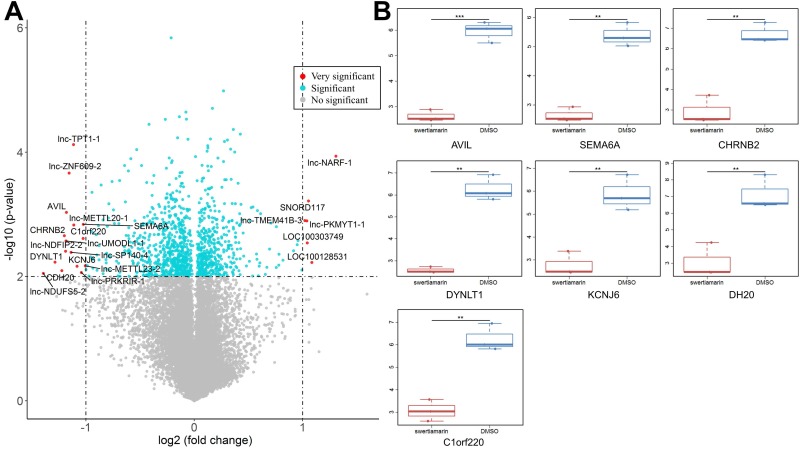
** The differential expressed genes in HepG2 cells treated by STM.** A. Differential expressed genes (DEGs) identified in STM treated HepG2 cells. The x-axis is log2 ratio of gene expression levels between two cells; the y-axis is p value based on -log10. The red dots represent the very significantly DEGs (P<0.01 and absolute value of log2(FC)>1 ); the blue dots represent the other DEGs (P<0.01 and absolute value of log2(FC)<=1 ); the gray blue dots represent the transcripts whose expression levels did not reach statistical significance (P>0.01). B. PCR analysis of seven DEGs.

To reveal the molecular mechanism by which STM inhibits the malignant phenotypes of HepG2 cells, we performed microarray analysis to screen differential genes in HepG2 cells exposed to STM. Compared to cells exposed to vehicle control, XXX genes were significantly upregulated and XXX genes were significantly downregulated in HepG2 cells exposed to STM (FC >2, P < 0.01, and AUC =1, Fig. 2A, Supplemental Table 1). Among the most significantly downregulated genes we selected 7 genes for PCR verification, and the results showed good correlation with microarray data (Fig. 2B).

### Pathway and biological process analysis of differentially expressed genes

**Figure 3:**
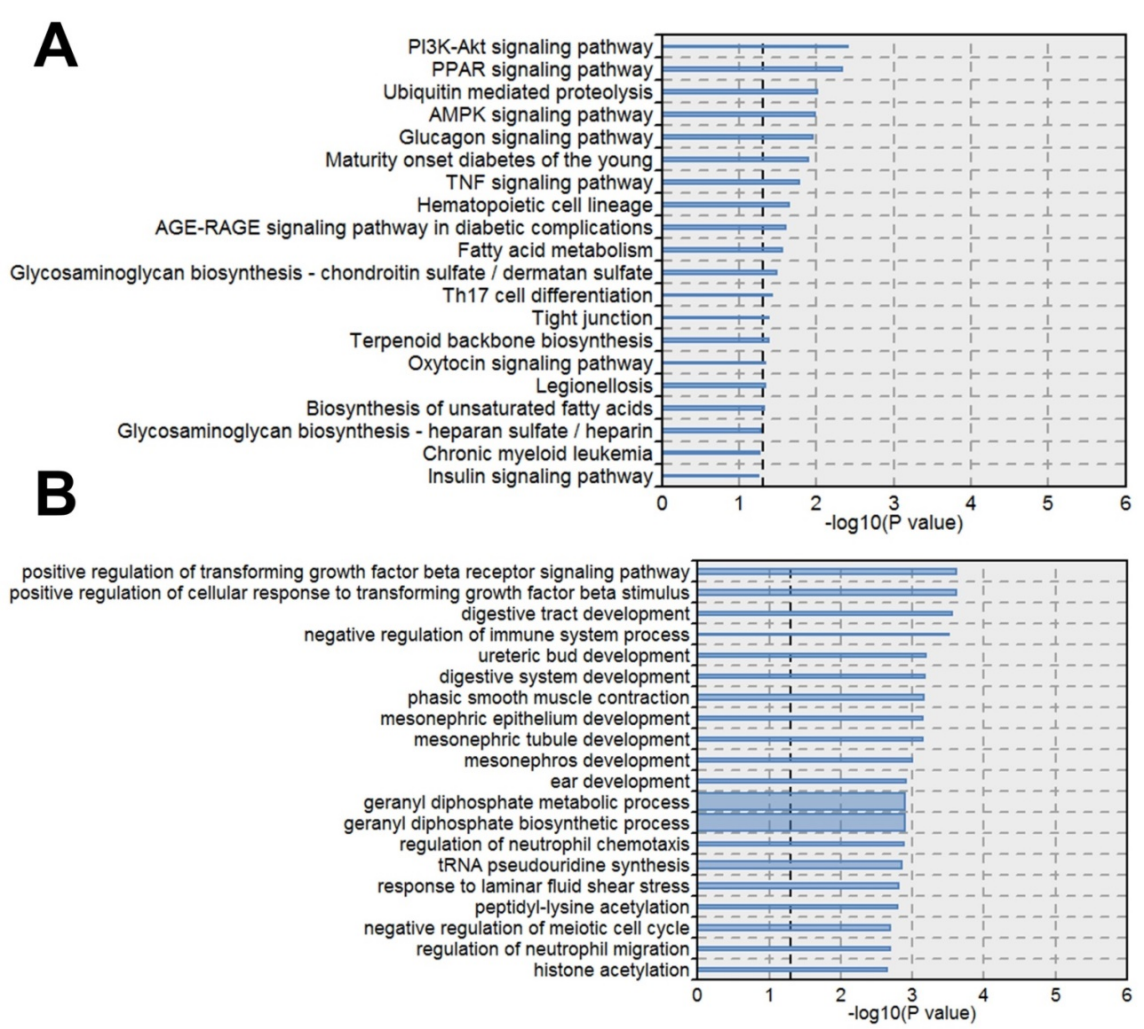
** Pathways and biological processes significantly enriched by differential expressed genes**. A. KEGG pathways enriched DEGs. B. Biological Processes enriched DEGs. The line width indicates the enrichment percentage. The dotted line in the box indicates the significance threshold (P value = 0.05).

**Figure 4:**
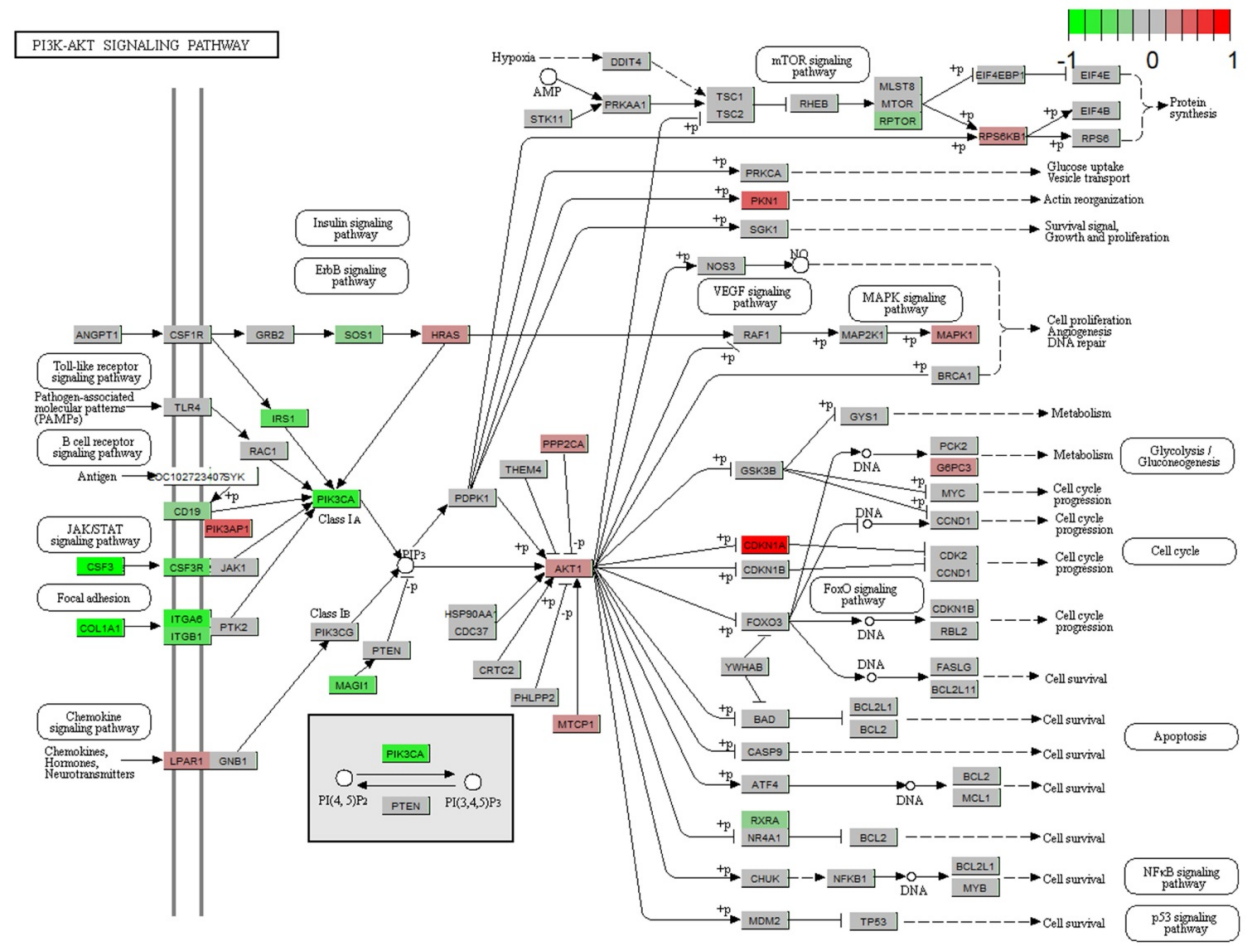
** Gene expression profiles of the PI3K-AKT signaling pathway in treated HepG2 cell.** The red and green colors represent the log2(FC) of the corresponding genes.

Next we performed pathway analysis to identify significant pathways of differentially expressed gene (DEGs) in accordance with KEGG (Fig. 3A). We also classified the DEGs into different biological processes (Fig. 3B). Among the significant pathways of DEGs, PI3K-Akt signaling pathway showed the highest P value, thus we further analyzed the expression changes of the DEGs involved in PI3K-Akt signaling pathway. The DEGs upregulated by STM were indicated by red while those downregulated by STM were indicated by green (Fig. 4).

### Prediction of targets of STM

**Figure 5:**
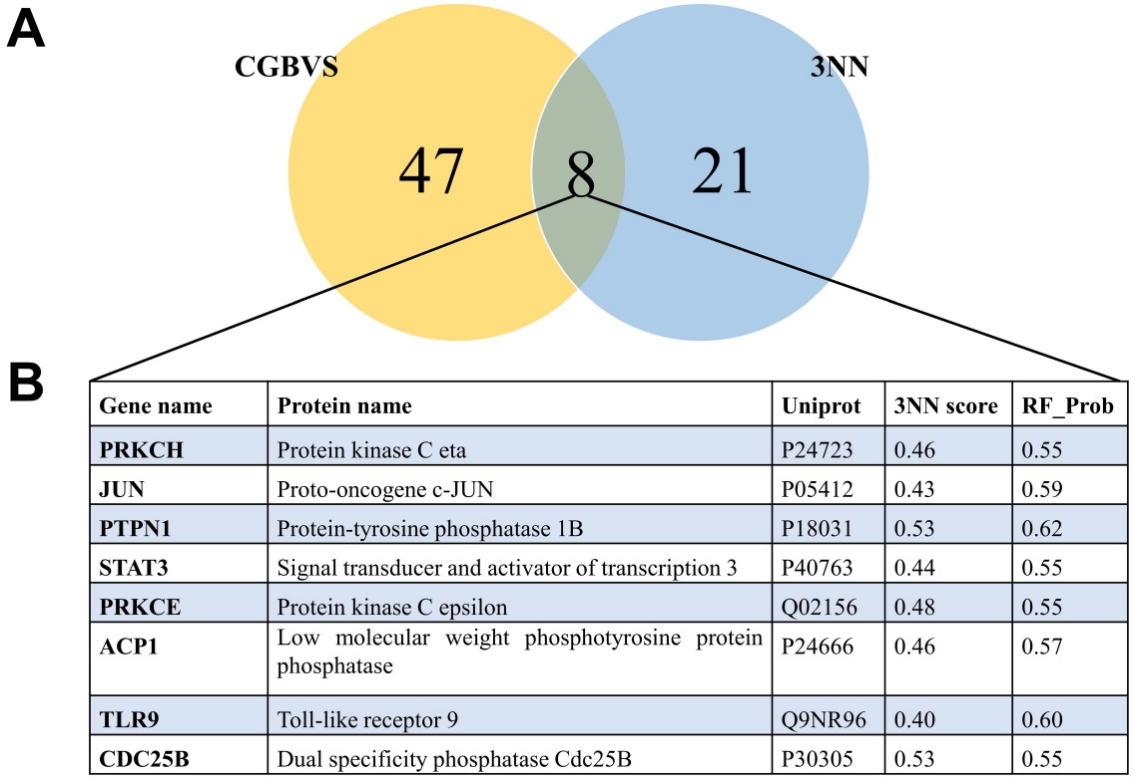
** Venn diagram of the predicted targets by two different methods and the consistent targets.** A. Venn diagram of the predicted targets by CGBVS and 3NN. B. Eight targets predicted by the two methods.

**Figure 6:**
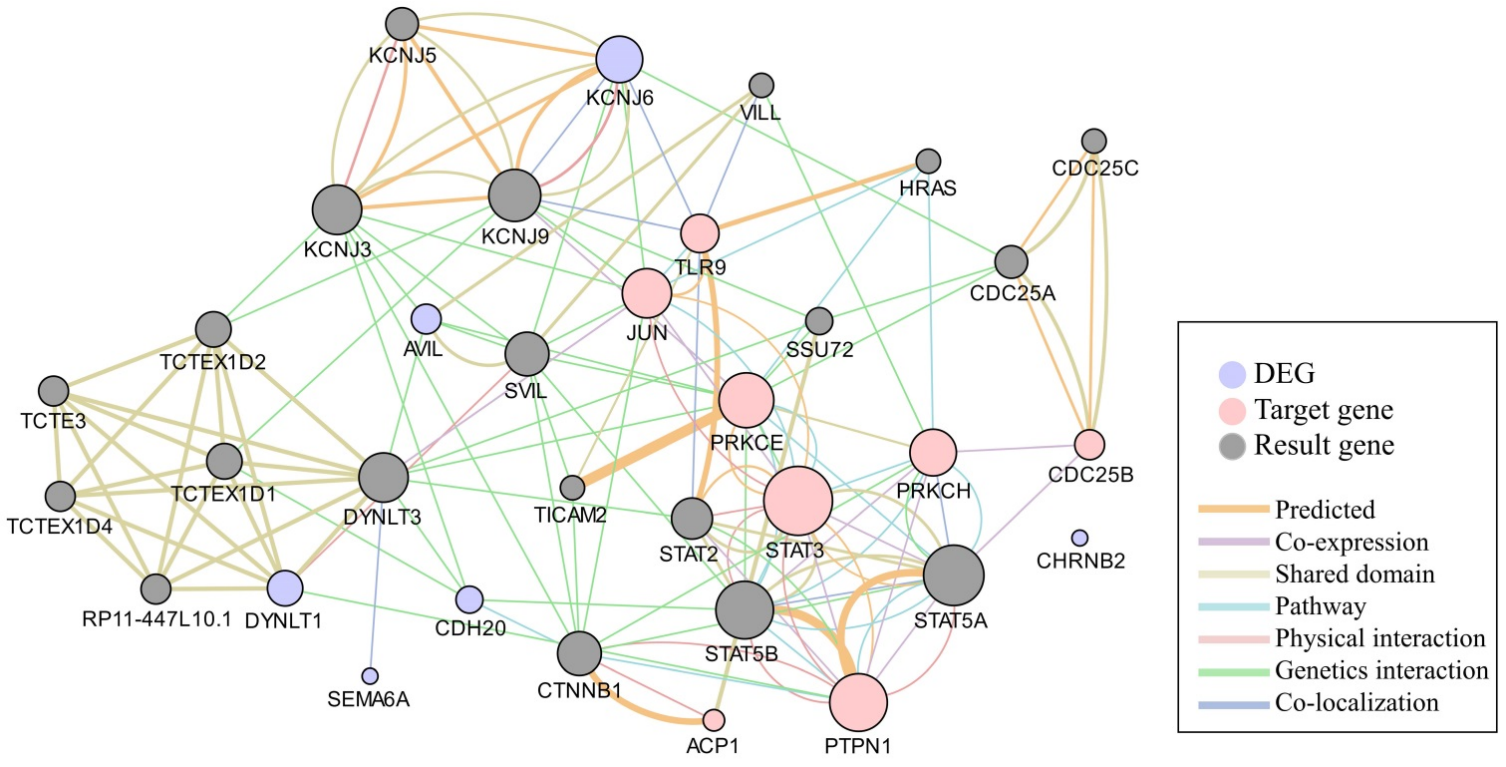
** Network analysis of the consistent targets and the most differential expressed genes.** The network shows the predicted functional interaction between the consistent targets (pink circle) and most DEGs (purple circle) according to the Genemania database. The gray nodes represents the result genes involved in the functional interaction between the pink and purple circles. Each colored line represents a different interaction, and the color line width indicates the confidence of the interactions.

To understand the mechanism underlying the anti-cancer activity of STM on HepG2 cells, we predicted the targets of STM. Chemical genomics-based virtual screening (CGBVS) could predict compound-protein interactions (CPIs) by using a support vector machine 8. K Nearest Neighbor with K=3 (3NN) is another powerful program to predict and characterize drug-target associations 9. We found that 47 targets of STM were predicted by CGBVS while 21 targets of STM were predicted by 3NN. Notably, 8 targets were predicted as the targets of STM by both programs (Fig. 5). Network analysis of these 8 targets and the most DEGs showed the potential functional interaction between them, which may then regulate the result gene (effector gene) to execute the activity of STM (Fig. 6).

### Regulation network of JUN and STAT3

**Figure 7:**
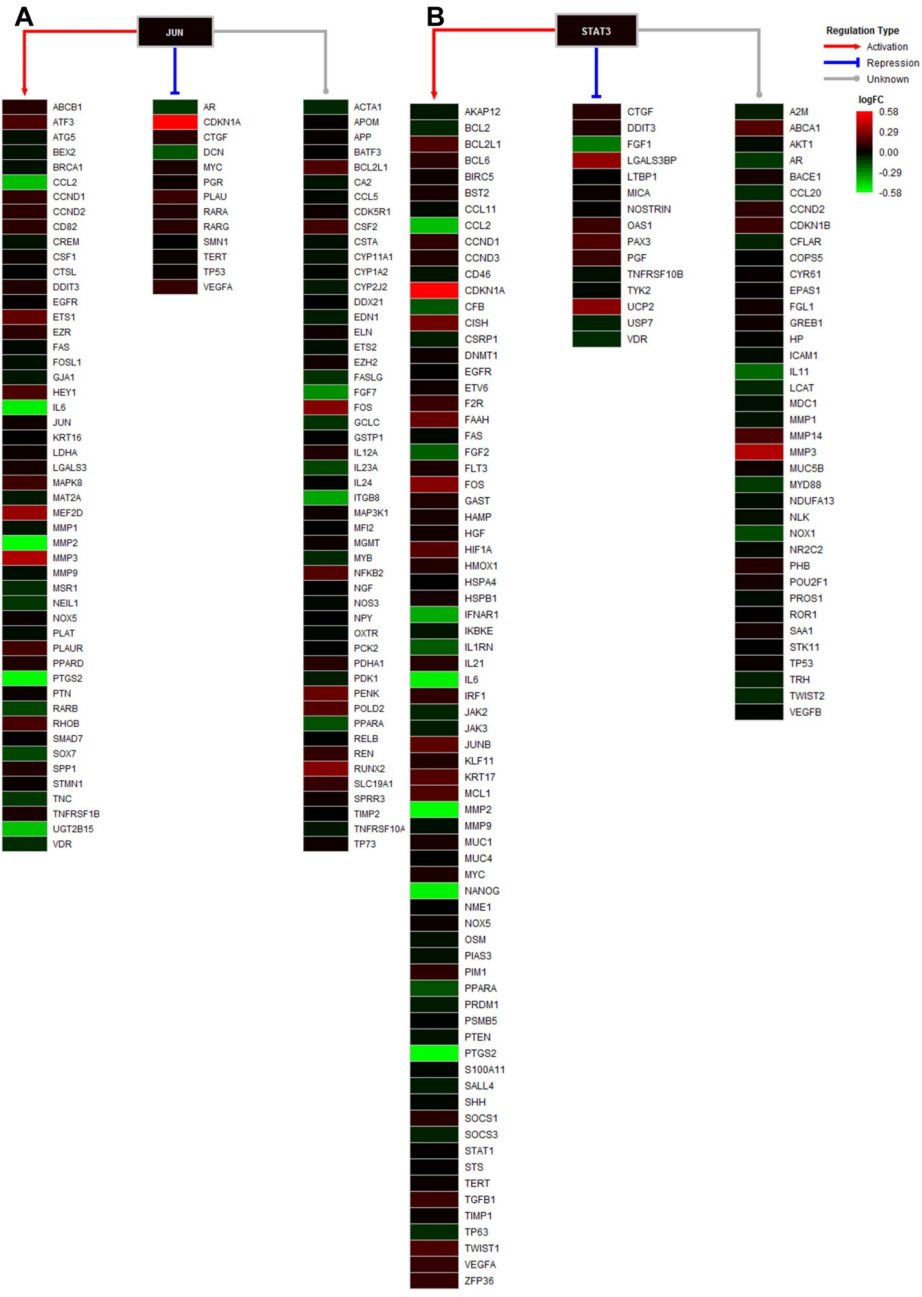
** Regulation analysis of transcription factors JUN and STAT3.** A. Regulatory model of JUN in the treated cell, including the regulation types of activation, repression, and unknown. B. Regulatory model of STAT3 in the treated cell, including the regulation types of activation, repression, and unknown. The gradient color from red to green is expressed as the logFC value of each gene.

Among the 8 predicted targets of STM, JUN and STAT3 are two important transcription factors known to regulate the expression of a wide variety of downstream target genes. Therefore, we performed network analysis of the DEGs whose expression is regulated by JUN and STAT3. We found that a large range of DEGs with different expression changes induced by STM could be regulated by JUN and STAT3 (Fig. 7).

## Discussion

STM is a secoiridoid glycoside mainly isolated from Enicostemma species and has demonstrated a wide variety of biological activities such as anti-inflammatory, anti-pyretic, anti-microbial, anti-malarial, and anti-diabetic activity 10-16. However, the anti-tumor activity of STM has seldom been reported. In this study, we first evaluated the anti-tumor activity of STM on HCC cells. Next we explored gene expression profiling of HepG2 cells treated by STM and performed bioinformatics analysis to elucidate the molecular mechanism and identify potential targets of STM to inhibit HCC.

Cell proliferation is known to be crucially regulated by cell cycle control. Chemoradiotherapy agents mainly inhibit tumor growth via the regulation of the expression and function of cell cycle regulatory proteins 17,18. Notably, a previous study reported that STM inhibited the proliferation and increased the apoptosis of fibroblast-like synoviocytes and this was related to the upregulation of caspase 3, an important effector of apoptosis, by STM in fibroblast-like synoviocytes 19. These findings are consistent with our data that STM inhibited the proliferation and increased the apoptosis of HepG2 cells. However, we did not find caspases in the list of DEGs. Instead, we found that BCL2L13 (BCL2-like 13), an apoptosis facilitator, was upregulated by STM in HepG2 cells. In addition, we found that cyclin-dependent kinase inhibitor 1A (CDKN1A, also known as p21), cyclin-dependent kinase inhibitor 1B (CDKN1B, also known as p27), cyclin-dependent kinase inhibitor 1C (CDKN1C, also known as p57) were all upregulated by STM in HepG2 cells (please see supplemental table 1). Collectively, these results suggest that STM inhibits HCC cell growth via the blockage of cell cycle progression and the induction of cell apoptosis.

MMPs are crucially involved in the degradation of extracellular matrix and facilitate tumor invasion and metastasis. STM has been shown to downregulate the expression of MMP1 and MMP3 in fibroblast-like synoviocytes 19. In this study, we did not find MMP1 and MMP3 in the list of DEGs. Instead, we found that MMP2 expression was downregulated by STM in HepG2 cells. Thus we speculate that STM may mainly downregulate MMP2 to inhibit the invasion ability of HepG2 cells. The differential regulation of MMPs by STM in different cell types may be due to cell type specificity. Further studies are needed to reveal the anti-metastasis mechanism of STM in HCC.

Using two different programs CGBVS and 3NN, we identified 8 targets of STM that were predicted by both programs. Among the 8 predicted targets of STM, JUN and STAT3 attracted our attention because they regulate the expression of a wide variety of downstream target genes which could modulate cell proliferation, apoptosis and invasion 20-22. CDKN1A expression is known to be repressed by JUN but its expression was significantly upregulated by STM in HepG2 cells (please see CDKN1A in bright red in Fig. 7A). On the other hand, MMP2 expression is known to be activated by JUN but its expression was significantly downregulated by STM in HepG2 cells (please see MMP2 in bright green in Fig. 7A). These results indicate that JUN is a target of STM and its activity is inhibited by STM. Similarly, MMP2 expression is known to be activated by STAT3 but its expression was significantly downregulated by STM in HepG2 cells (please see MMP2 in bright green in Fig. 7B). In addition, STM attenuated inflammation via inhibiting JAK2/STAT3 signaling in adjuvant induced arthritis 23. STAT3 is known to be activated to regulate cell survival and apoptosis in response to stress 24,25. Therefore, STAT3 is another target of STM and its activity is inhibited by STM. Moreover, STW was shown to attenuate carbon tetrachloride-induced liver fibrosis via the inhibition of TGFß1/SMAD pathway 26. In addition, a recent study revealed a common pathway that regulated both MMPs and Smad4 in vulvar squamous cell cancer 27. While we did not find SMAD, TGFß1 was shown in Fig. 7B. Next we will perform functional studies to verify the predicted targets of STM, which will explain how STM exerts anti-tumor efficacy in HCC.

In conclusion, STM treatment led to significant changes in the expression of a variety of genes that modulate cell survival, cell cycle progression, apoptosis, and invasion. Moreover, most of these genes can be clustered into pathway networks such as PI3K, JUN, STAT3, which are predicted targets of STM. Further confirmation of these targets will reveal the anti-tumor mechanisms of STM and pave the way for the development of STM as a novel agent for cancer prevention and treatment.

## Supplementary Material

Supplementary table.
